# Immunomodulatory effects of cysteamine and its potential use as a host-directed therapy for tuberculosis

**DOI:** 10.3389/fimmu.2024.1411827

**Published:** 2024-10-28

**Authors:** Saeid Najafi-Fard, Chiara Farroni, Linda Petrone, Anna Maria Gerarda Altera, Andrea Salmi, Valentina Vanini, Gilda Cuzzi, Tonino Alonzi, Emanuele Nicastri, Gina Gualano, Fabrizio Palmieri, Mauro Piacentini, Delia Goletti

**Affiliations:** ^1^ Translational Research Unit, National Institute for Infectious Diseases Lazzaro Spallanzani-Istituto di Ricovero e Cura a Carattere Scientifico (IRCCS), Rome, Italy; ^2^ Unità Operativa Semplice (UOS) Professioni Sanitarie Tecniche, National Institute for Infectious Diseases Lazzaro Spallanzani-Istituto di Ricovero e Cura a Carattere Scientifico (IRCCS), Rome, Italy; ^3^ Clinical Division of Infectious Diseases, National Institute for Infectious Diseases Lazzaro Spallanzani-Istituto di Ricovero e Cura a Carattere Scientifico (IRCCS), Rome, Italy; ^4^ Respiratory Infectious Diseases Unit, National Institute for Infectious Diseases Lazzaro Spallanzani-Istituto di Ricovero e Cura a Carattere Scientifico (IRCCS), Rome, Italy; ^5^ National Institute for Infectious Diseases Lazzaro Spallanzani-Istituto di Ricovero e Cura a Carattere Scientifico (IRCCS), Rome, Italy; ^6^ Department of Biology, University of Rome “Tor Vergata”, Rome, Italy

**Keywords:** cysteamine, tuberculosis, host-directed therapy, inflammation, Ag-specific response, PPD-specific response, apoptosis, necrosis

## Abstract

**Objective:**

Cysteamine, a drug approved to treat cystinosis, has been proposed as a host-directed therapy for *M. tuberculosis* (Mtb) and SARS-CoV-2. The impact of cysteamine on the immune responses has not been fully investigated. We aimed to *in vitro* evaluate the immunomodulatory effects of cysteamine on peripheral blood mononuclear cells (PBMCs) using the purified protein derivative (PPD) as a recall antigen, and an unspecific stimulus as staphylococcal enterotoxin B (SEB).

**Methods:**

PBMCs isolated from subjects with tuberculosis infection (TBI), those with tuberculosis disease (TB), and healthy controls (HC) were *in vitro* stimulated with PPD or SEB and treated or not with cysteamine at different concentrations (50 µM–400 µM) for 6 hours (h) and 24 h. We evaluated the T helper1 (Th1) and T cytotoxic1 (Tc1) cell cytokine production by flow cytometry and immune-enzymatic assays. In HC, we also evaluated apoptosis and/or necrosis by flow cytometry.

**Results:**

We observed an immunomodulatory effect of cysteamine at 400 µM in PBMCs from TB and TBI subjects. It significantly reduced PPD-specific Th1 responses at 24 h and at 6 h (p=0.0004 and p=0.0009, respectively), and a similar non-significant trend was observed with cysteamine at 200 µM (p=0.06 at 24 h and p=0.14 at 6 h). Moreover, cysteamine at both 400 µM (p<0.0001 and p=0.0187 at 24 h, respectively, and p<0.0001 at 6 h for both) and 200 µM (p=0.0119 and p=0.0028 at 24 h and p=0.0028 and p=0.0003 at 6 h, respectively) significantly reduced SEB-induced Th1 and Tc1 responses. Furthermore, we found that cysteamine induced morphological lymphocyte changes and significantly reduced the lymphocyte percentage in a dose- and time-dependent manner. Cysteamine at 400 µM induced 8% late apoptosis and 1.6% necrosis (p<0.05) at 24 h. In contrast, despite significant differences from untreated conditions (p<0.05), cysteamine at 400 µM for 6 h induced approximately 1% late apoptosis and 0.1% necrosis in the cells.

**Conclusions:**

High doses of cysteamine *in vitro* reduce the percentages of PPD- and SEB-induced Th1 and Tc1 cells and induce late apoptosis and necrosis. Differently, cysteamine at lower doses retains the immunomodulatory effect without affecting cell viability. These findings suggest cysteamine as a potential adjunct to antimicrobial regimens as in the TB or COVID-19 field, for its ability to reduce the inflammatory status.

## Introduction

Tuberculosis (TB), caused by *Mycobacterium tuberculosis* (Mtb), is one of the most devastating infectious diseases worldwide with more than 10 million new cases and 1.3 million deaths per year (1). Mtb mainly infects the lungs and causes immune activation, inflammation, and the formation of granulomas, which is the hallmark of TB disease (1, 2). Monocyte-derived macrophages, foamy macrophages, epithelioid cells, and multinucleated giant cells can contain mycobacteria within granulomas and produce proinflammatory cytokines and chemokines. They can also present antigens to T cells that can recruit additional cells and kill intracellular mycobacteria once activated (3, 4). The unbalanced and persistent overactivated inflammatory pathways can lead to lung destruction and cavity formation (2, 5). While the current standard therapy for TB is effective, treatment challenges can occur either for non-adherence, failure, or drug resistance (1, 6). Moreover, the long duration associated with TB treatment may contribute to reduced patient compliance. A better understanding of host–pathogen interactions and the development of novel interventions, such as host-directed therapies, may significantly improve the patients’ management even in the case of drug resistance (7). Anti-inflammatory agents, by targeting the host rather than the pathogen, have been suggested as adjunctive host-directed therapies for their potential to modulate lung inflammation (8, 9) and in TB, to shorten treatment duration (5). In this regard, the repurposing of drugs approved for treating diseases different from TB may be used in the field of infectious diseases (10). For instance, thiol-containing compounds have been demonstrated to have potent biological effects, for protecting from bacterial (11) and viral infections (12, 13). An example of these compounds is cysteamine (2-mercaptoethylamine or aminoethanethiol), an aminothiol synthesized during the degradation of coenzyme A (14) used for the treatment of cystinosis, which is an autosomal recessive lysosomal storage disease (15). Cysteamine has several biological effects influencing the oxidative state of the cells and regulating pathways involved in cellular homeostasis (16) and inflammation (17, 18). For these pleiotropic functions, cysteamine has been suggested as a possible treatment for several diseases with different etiology such as the neurodegenerative disorder Huntington disease (HD) (19, 20), major depressive disorder (21), asthma (22), non-alcoholic fatty liver disease (NAFLD) (23), Alzheimer’s disease (AD), and Parkinson’s disease (PD) (24). Intriguingly, we and others have shown anti-infective properties of cysteamine against Mtb (25–27), *Pseudomonas aeruginosa* (28), *Plasmodium* species (29), human immunodeficiency virus type 1 (HIV-1) (30, 31), influenza A virus H1N1 (32), and, more recently, SARS-CoV-2 (13, 33, 34) infections. Moreover, cysteamine acts as a pharmacological inhibitor of transglutaminase 2 (TG2) (18, 35), a ubiquitous enzyme involved in several crucial cellular processes such as cell death/survival and autophagy (36). TG2 may also play a key role in the pathogenesis of Mtb infection (25), but not in SARS-CoV-2 (13). Indeed, it was shown that *in vitro* pharmacological inactivation of TG2, e.g., with cysteamine, enhances the anti-mycobacterial properties of Mtb-infected macrophages, correlating with reduced cell death and impaired autophagy homeostasis (25, 26). In COVID-19, we and others showed that cysteamine may down-modulate the hyperinflammation caused by SARS-CoV-2 (13, 33, 34). In addition, TB, similarly to COVID-19, can cause tissue damage resulting from sustained inflammation leading to permanent pulmonary disability. As a result, decreasing inflammation can protect the lungs from harm and improve the effectiveness of TB treatment (37).

However, so far, the potentials of cysteamine as host-directed therapy have not been investigated in detail and the knowledge of the impact of cysteamine in Mtb-specific immune responses is scarce. In this context, the study of the immune response, particularly the specific T response, is crucial. Thus, we evaluated the role of cysteamine on the ability to modulate the *in vitro* Mtb-recall antigen response in peripheral blood mononuclear cells (PBMCs) from subjects with TB or TB infection (TBI) or healthy controls (HC) vaccinated with Bacillus Calmette et Guerin (BCG). We showed that cysteamine decreases the Mtb-specific response of T cells in a time- and dose-dependent manner; in particular, it decreases interferon (IFN)-γ, tumor necrosis factor (TNF), and interleukin (IL)-2-specific response, which are master drivers of inflammation in TB.

## Methods

### Study population

This study was approved by the Ethical Committee of the National Institute of Infectious Diseases Lazzaro Spallanzani IRCCS (approval number 72/2015) and conducted between 2019 and 2023. Written informed consent was required to participate in the study. TB patients (n=12) and TBI subjects (n=10) were enrolled in the study. Patients with TB disease were included if diagnosed with drug-susceptible pulmonary TB (positive culture or molecular tests for Mtb) and enrolled within 7 days of starting TB treatment. TBI was defined based on a positive score to QuantiFERON Gold Plus (QTF-Plus) (38–40) or radiological findings, indicating a previous Mtb infection in the absence of clinical, microbiological, and radiological signs of TB disease. Moreover, HC (n=19, of whom 10/19 were BCG-vaccinated) who scored negative on QTF-Plus test were used as healthy controls (**Table 1**). The precise number of subject samples involved in each experiment is detailed in the respective paragraph within the Methods section and in the figure legends.

**Table 1:** Demographic and clinical characteristics of the enrolled subjects.

	TB	TBI	HC	TOTAL	p value
**N (%)**	12 (29)	10 (24)	19^†^(46)	41 (100)	
**Age median (IQR)**	43.5 (35.25-62.25)	48.5 (39.75-56)	50 (39-54)	46 (38-55)	0.96 ^#^
**Male N (%)**	12 (100)	3 (25)	7 (37)	22 (54)	**0.0006** ^§^
Origin N (%)					
** Western Europe**	3 (25)	5(50)	17 (89)	25 (61)	**0.0007** ^§^
** Eastern Europe**	7 (58)	1 (10)	–	8 (20)	
** Asia**	–	1 (10)	–	1 (2)	
** Africa**	2 (16)	1(10)	–	3 (7)	
** South America**	–	2 (20)	2 (11)	4 (10)	
**BGC-vaccinated N (%)**	9 (75)	2 (50) *	11 (0) **	15 (62)	
QFT-Plus results N (%)					
** Positive**	7 (58)	9 (90)	–	16 (39)	
** Negative**	2 (17)	1 (10)	19 (100)	22 (54)	
** Not available**	3 (25)	–	–	3 (7)	

**Figure 1:**
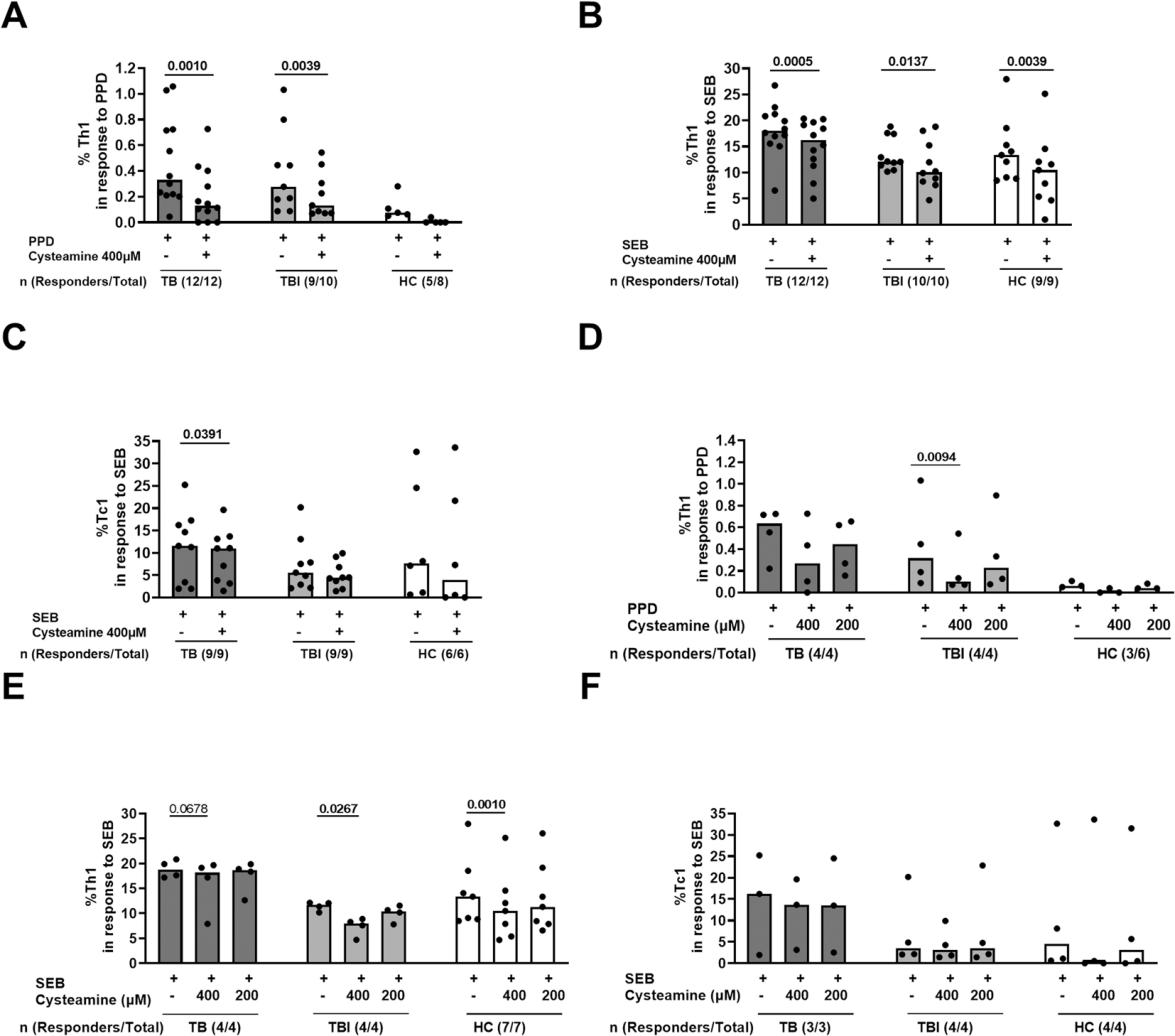
The effect of cysteamine on the specific Th1 and Tc1 responses in TB and TBI subjects. PBMCs from TB (n=12), TBI (n=10), and HC (n=9) were stimulated *in vitro* for 24 h and treated with cysteamine at 400 µM **(A–C)** and, in a subgroup of subjects, also with 200 µM **(D–F)**. **(A, D)** By flow cytometry, the CD4^+^ Th1-specific response was evaluated in PPD- and **(B, C, E, F)** the CD8^+^ Tc1-specific response in SEB-stimulated cells. The total number of each group are reported as evaluable totals. Statistical analysis was performed using Wilcoxon matched-pairs rank test. Significant p values are indicated in bold. Data are reported as median, and each dot represents a different individual.

TB, tuberculosis disease; TBI, tuberculosis infection; HC, healthy controls; N, number; IQR, interquartile range; QFT-Plus, QuantiFERON; BCG, bacillus Calmette-Guérin. # Kruskal-Wallis test. § Chi-square. *Data available for 8/10 subjects. **Data available for 18/19 individuals. ^†^Among HC: 9 individuals were evaluated in the first experiments shown in **Figure 1**. These HC were enrolled together with the TB and TBI cohorts. Differently, the remaining 10 HC were evaluated in the cohort used to assess the cell viability and Th1, Tc1, and IL-10 response. The bold values indicate statistically significant differences.

Exclusion criteria for enrollment were age ≤18 years, infection with HIV, HCV, HBV, or being immunocompromised or immune suppressed. The study complied with the principles of the Declaration of Helsinki.

### Cysteamine preparation and medium

Cysteamine was purchased as a powder (CAS 60-23-1; Merck Life Science, Milan, Italy; Cat. No. M9768) and prepared in deionized water at a concentration of 200 mM as a stock. Then, the solution was filtered and stored for a maximum of a week at 4°C protected from light. At the time of each experiment, the prepared cysteamine solution was diluted in the complete medium [RPMI-1640 (Gibco, CA, USA), 10% fetal bovine serum (FBS) (Gibco, Life Technologies Italia, Monza, Italy), 2 mM L-glutamine, and 1% penicillin/streptomycin] before being added to the cells (below are detailed the working concentrations) (33).

### PBMCs stimulation for flow cytometry analysis

Briefly, PBMCs were isolated from the blood of enrolled subjects using Ficoll density gradient centrifugation. For the *in vitro* stimulation and treatment, PBMCs were seeded in a complete medium, and 10^6^ cells/mL/condition were stimulated with or without purified protein derivative (PPD) at 10 µg/mL or staphylococcal enterotoxin B (SEB) (Merck Life Science Cat. S4881) at 200 ng/mL (positive control) with α-CD28 and α-CD49 monoclonal antibody (mAb) (1 µg/mL each) (BD Biosciences, San Jose, USA) when appropriate. Cells were then treated or not with cysteamine at different concentrations (400 µM, 200 µM, 100 µM, or 50 µM) in complete RPMI-1640. BD Golgi Plug was added when appropriate after 1 h of stimulation, and cells were incubated for 6 hours (h) or 24 h at 37°C, 5% CO_2_. Supernatants were collected and stored at −80°C until further use.

### Cytokine detection

The levels of IFN-γ, IL-2, TNF, and IL-10 were measured in supernatants from stimulated PBMCs using the ELLA Simple Plex Human Assay (Bio-Techne, Minneapolis, MN, USA, Cat. SPCKC-PS-003978 customized kit and Cat. SPCKB-PS-000276) that is a fully automated system based on a ready-to-use cartridge. Stimulated sample values were subtracted from the respective control. The ranges were: 0.17 pg/mL–4,000 pg/mL for IFN-γ, 0.54 pg/mL–2050 pg/mL for IL-2, 0.3 pg/mL–1160 pg/mL for TNF, and 0.58 pg/mL–2212 pg/mL for IL-10.

### Intracellular staining and flow cytometry analysis

For preliminary experiments, cells were stained with ACQUA DYE-AmCyan (Invitrogen), CD3 PE-Cy7, CD8 Pacific Blue (all from BD), and CD4 PerCP-Vio700 (Miltenyi) for 20 min at 4°C, fixed with formalin 4% for 5 min at room temperature, and then stained for 20 min at 4°C for intracellular cytokines IFN-γ APC, TNF FITC, and IL-2 PE in PBS + 2% FCS + saponin 0.5%. Cells from BGC-vaccinated HC were washed with PBS and stained in Brilliant Stain Buffer (BD) for surface markers: Fixable Viability Stain 700 (FVS700), CD3 PE-Cy7, CD8 Pacific Blue (all from BD), and CD4 ECD (Beckman Coulter). Cells were washed and fixed/permeabilized with Cytofix/Cytoperm (BD) according to the manufacturer’s instructions. After that, cells were intracellularly stained for 30 min at 4°C in the dark with IFN-γ APC, TNF FITC, and IL-2 PE. At least 100,000 lymphocytes were acquired using CANTO II (BD) or DxFLEX (Beckman Coulter) cytometers and analyzed with FlowJo software (version 9.3.2 and version 10.8.1). The lymphocyte population was gated according to the forward (FSC-A) and side scatter (SSC-A) panels, and then the singlets were gated based on FSC height (FSC-H) and area (FSC-A). CD4^+^ and CD8^+^ T-cells were gated within the CD3^+^ live T-cell subset according to the negativity of the Fixable Viability Stain 700 antibody (LD). The cytokine responses were scored positive if the percentage of the PPD- or SEB-stimulated cells was at least twofold higher compared with the unstimulated control (41, 42). T helper type 1 (Th1)-specific response refers to the CD4^+^ T cells producing any of the following cytokines IFN-γ and/or IL-2 and/or TNF. T cytotoxic type 1 (Tc1)-specific response refers to CD8^+^ T cells producing any of the cytokines IFN-γ and/or IL-2 and/or TNF (43, 44). The complete gating strategy is shown in **Supplementary Figure S1**.

### Cell apoptosis and necrosis evaluation

PBMCs were stained with Annexin-FITC and Propidium Iodide-PE according to manufacturer instructions (kit ref. IM3546, Beckman Coulter). Samples were acquired (250,000 events gated as all events) using a DxFLEX cytometer (Beckman Coulter) and analyzed with FlowJo software (version 10.8.1). Gates were established based on the unstimulated and untreated cells. The analyses were conducted blindly by two different operators (CF and SNF) to avoid any bias. The gating strategy is shown in **Supplementary Figure S5**. Cells negative for both markers are considered as live cells, cells positive for Annexin V only are considered as in early apoptosis, cells positive for both Annexin V and PI are cells in late apoptosis, and cells positive for PI only were considered as in necrosis.

### Statistical analysis

Data were analyzed using GraphPad (GraphPad Prism V.8.0.1). Continuous and categorical variables were reported, respectively, as median and IQR. Wilcoxon test and Friedman test for paired data were used. Dunn’s multiple p-value correction test was applied when several groups were compared. Categorical variables were analyzed by the chi-square test; the Kruskal–Wallis test was used for comparison among several groups. Two-tailed p values were considered significant if <0.05, except for those analyses where a correction was applied.

## Results

### Clinical and demographical characteristics of the enrolled subjects

We enrolled 12 TB, 10 TBI, and 19 HC. The clinical and demographical characteristics of the enrolled subjects are reported in **Table 1**. For the initial setting of experiments, among the HC group, nine subjects were enrolled together with the TB and TBI cohorts. In these subjects, we evaluated the effect of cysteamine on the specific Th1 and Tc1 responses, as shown in **Figure 1**. The remaining 10 BCG-vaccinated HC were enrolled to assess the effect of cysteamine on the cell viability as well as Th1, Tc1, and IL-10 responses in a time- and dose-dependent manner (**Table 1**). Among the groups, no significant differences were found for age (p=0.96), whereas we found significant differences for gender (p=0.0006) and origin (p=0.0007). Among the TBI individuals, one subject scored negative to the QFT-Plus but was included in the TBI group based on the presence of radiological scars in the upper lung lobes (see Material and Methods section).

### Cysteamine modulates T cell-specific responses in TB and TBI subjects

In a preliminary study, we evaluated the immunomodulatory effect of cysteamine on T cells and T cell-specific response in subjects with TB disease (n=12), TBI (n=10), and HC (9/19) (**Table 1**). We used PPD, as a surrogate for mycobacterial antigen stimulation. The Th1-specific response was evaluated in PBMCs stimulated with PPD and treated with cysteamine at 400 µM. Cysteamine significantly reduced the Th1-specific response in both TB and TBI compared with the untreated sample (p=0.001 and p=0.0039, respectively). Similarly, cysteamine significantly reduced the Th1 response to SEB (TB, p= 0.0005; TBI, p= 0.0137, HC, p=0.0039) (**Figures 1A, B**). On the other hand, Tc1 response to SEB was significantly reduced only in the TB group (p=0.0391) (**Figure 1C**). No PPD-specific Tc1 response was detected at either time point (**Table 2**). In a subgroup of individuals, we tested cysteamine at two different concentrations, 400 µM and 200 µM. For HC, responses to PPD were found only in three subjects. Despite the small sample size, we showed that cysteamine at 400 µM reduced PPD-induced CD4^+^ Th1 response. This reduction was statistically significant only within the TBI group (p=0.0094) (**Figure 1D**). Moreover, cysteamine at 400 µM reduced SEB-induced Th1 response within TB (although not significant, p=0.068), and in a statistically significant manner within TBI (p=0.027), and HC (p=0.001) (**Figure 1E**). Although not statistically significant, a reduced trend of the PPD-specific response was observed after treatment with cysteamine at 200 µM in all groups (**Figure 1D**). We did not observe significant differences in the specific Tc1 response induced by SEB in any of the three groups evaluated (**Figure 1F**).

**Table 2:** The effect of cysteamine on T cells responses as Th1 and Tc1 cytokines production by flow cytometry.

		PPD	PPD+ cysteamine 400 µM	p value^§^	PPD+ cysteamine 200 µM	p value^#^	SEB	SEB+ cysteamine 400 µM	p value^§^	SEB+ cysteamine 200 µM	p value^#^
**6 h**	%Th1	0.23 (0.13-0.69)	0.04 (0.005-0.16)	**0.0009**	0.16 (0.09-0.31)	0.14	9.50 (6.1-11.2)	4.8 (2.2-7.5)	**<0.0001**	7.6 (5.0-9.5)	**0.0028**
	%Tc1	–	–	–	–	–	7.3 (4.0-10.1)	3.5 (2.0-6.2)	**<0.0001**	4.5 (2.7-7.0)	**0.0003**
**24 h**	%Th1	0.24 (0.17-0.51)	0.07 (0.03-0.20)	**0.0004**	0.2 (0.10-0.37)	0.06	15.5 (9.9-19.0)	10.3 (6.5-13.9)	**<0.0001**	11.7 (7.4-15.2)	**0.0119**
	%Tc1	–	–	–	–	–	7.0 (3.7-9.7)	4.2 (3.4-7.3)	**0.0187**	3.8 (2.7-7.8)	**0.0028**

PPD, purified protein derivative of tuberculin; SEB, staphylococcal enterotoxin B; h, hours; Th1, T helper type 1; Tc1, T cytotoxic type 1. §, # Friedman test with Dunn’s multiple correction between PPD (or SEB) vs. PPD (or SEB) + cysteamine 400 µM and PPD (or SEB) vs. PPD (or SEB) + cysteamine 200 µM, respectively. The bold values indicate statistically significant differences.

### Cysteamine modulates T-cell responses in a dose-dependent manner

**Figure 2:**
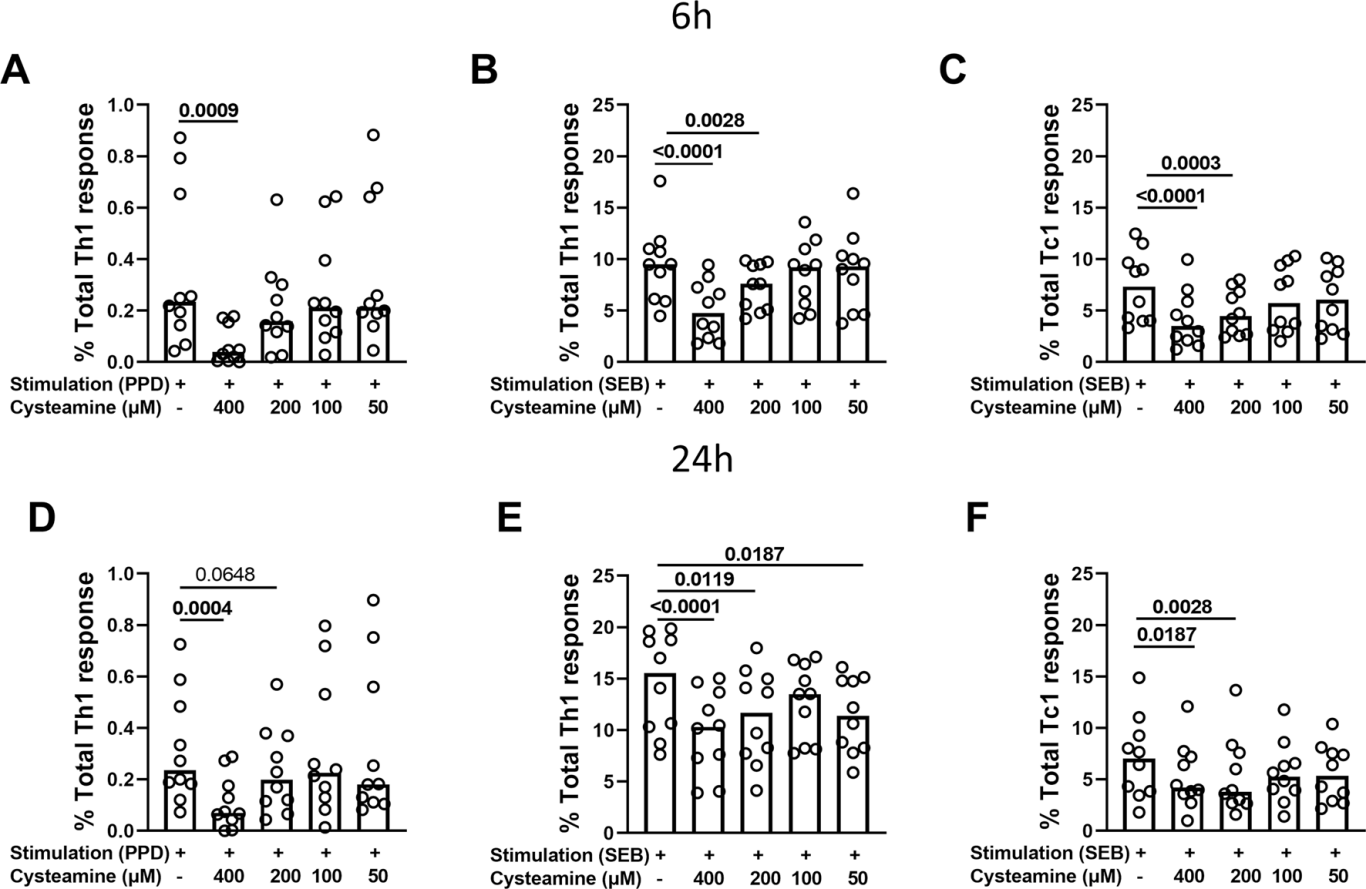
Evaluation of Th1- and Tc1-specific responses after treatment with cysteamine in HC BCG-vaccinated individuals. PBMCs from BCG-vaccinated HC (n=10) were stimulated with **(A–D)** PPD and **(B, C, E, F)** SEB for 6 h or 24 h, as indicated in each graph. Cells were treated or not with cysteamine at different concentrations (50 µM–400 µM), and the percentages of Th1- and Tc1-specific response were evaluated by flow cytometry. Each dot represents a single subject. Friedman test followed by Dunn’s multiple comparisons was performed, and significant p values are indicated in bold. Data are reported as median.

The immunomodulatory effects of cysteamine on antigen-specific Th1 and Tc1 responses were evaluated depending on the timing of stimulation. Hereafter, we performed the experiments only in the PBMCs from the BCG-vaccinated HC cohort (n=10) (**Table 1**). Cells were *in vitro* stimulated with either PPD or SEB and treated with different concentrations of cysteamine for 6 h or 24 h, and then the percentages of IFN-γ-, TNF-, and IL-2-producing CD4 (Th1) and CD8 (Tc1) cells were evaluated by flow cytometry. As shown in **Figure 2** and **Table 2**, a PPD-specific response was found only in the CD4^+^ T cells in all studied subjects at both time points, whereas no response was detected in the CD8^+^ T cells, as expected (42). Interestingly, no difference in the percentage of PPD-specific Th1 responses was found comparing the stimulation at 6 h and 24 h (data not shown). Cysteamine treatment at 400 µM significantly decreased the percentages of Th1 response to PPD at both time points (6 h: p=0.0009; 24 h: p=0.0004); a similar decreasing trend, although non-significant, was observed using cysteamine at 200 µM (**Figure 2**, **Table 2**). Moreover, cysteamine had similar modulatory effects in cells treated with SEB (**Figure 2**, **Table 2**). We did not observe any significant differences when cells were treated with cysteamine at lower doses (lower than 200 µM) (**Figure 2**) Thus, our *in vitro* data indicated that the T-cell responses are detectable after both 6-h and 24-h stimulation with either PPD or SEB and that cysteamine has an immunomodulatory effect, which is significant at the highest concentration.

### Cysteamine modulates cytokine production

**Figure 3:**
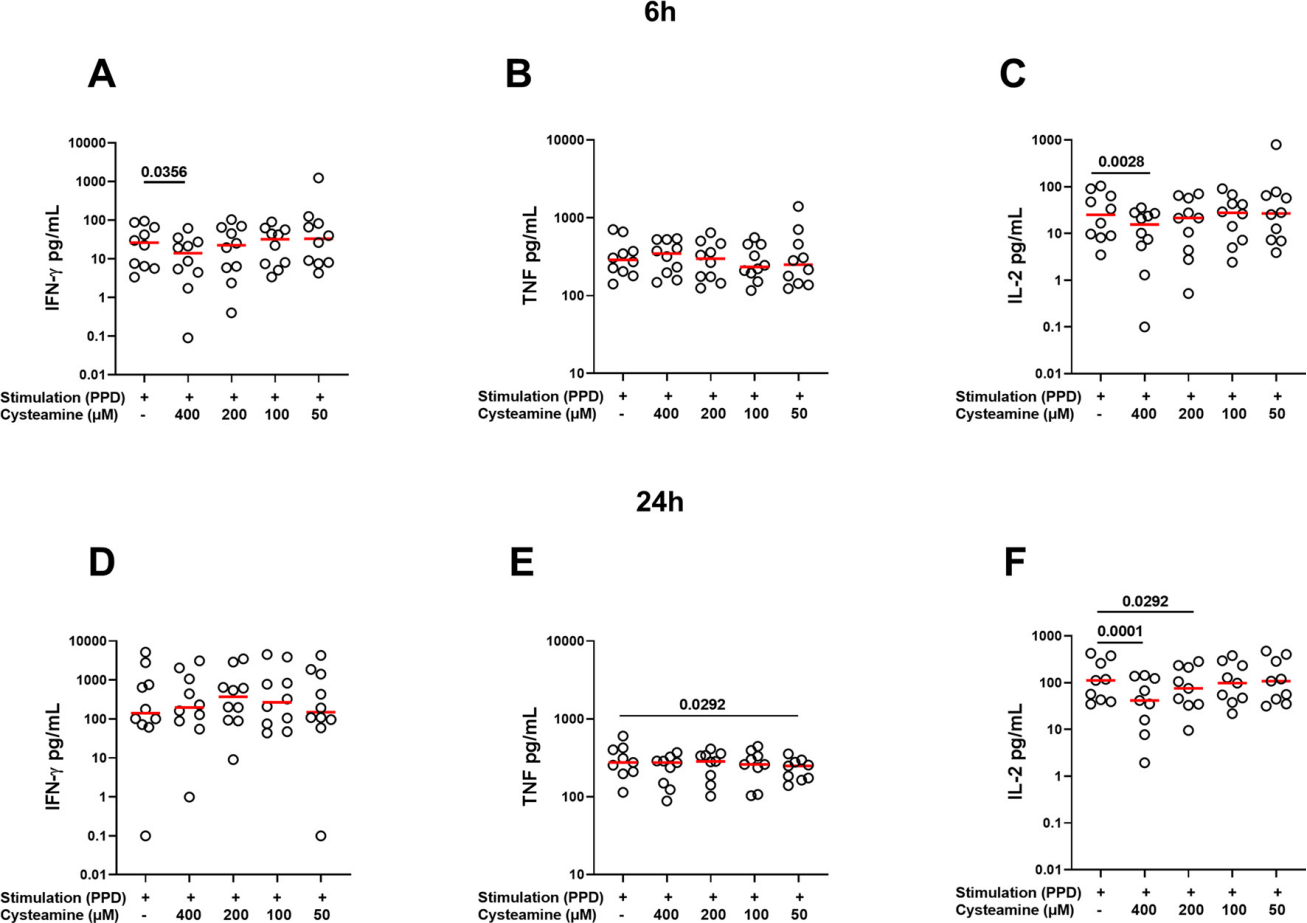
Treatment with cysteamine modulates the cytokine production. Cytokine production was evaluated in supernatants of PBMCs (BCG-vaccinated HC, n=10) stimulated *in vitro* with PPD for 6 h **(A–C)** and 24 h **(D–F)** and treated or not with cysteamine (50 µM–400 µM). Levels of **(A, D)** IFN-γ, **(B, E)** TNF, and **(C, F)** IL-2 were measured using an automated ELISA assay (ELLA). Statistical analysis was performed using Friedman test followed by Dunn’s multiple comparisons. Values from stimulated samples were subtracted from the respective unstimulated control. Red lines indicated the median, and each dot represents a different individual.

Then, we measured the levels of IFN-γ, TNF, IL-2, and IL-10 released by PBMCs stimulated for 6 h or 24 h with PPD or SEB and treated or not with cysteamine. The analysis was performed for all conditions in at least 9 of the 10 BGC-vaccinated HC. As shown in **Supplementary Table 1**, in response to 6 h PPD stimulation, cysteamine at 400 µM significantly reduced the IFN-γ and IL-2 production (p=0.0356 and p=0.0028, respectively), whereas TNF was not modulated regardless of the cysteamine concentration tested. Cysteamine at both 400 µM and 200 µM after 24 h of PPD stimulation significantly reduced IL-2 production (p=0.0001 and p=0.029, respectively), whereas TNF production was significantly reduced only when cells were treated with cysteamine at 50 µM (p=0.029). Differently, cysteamine did not modulate the IFN-γ levels at 24 h (**Figure 3**, **Supplementary Table 1**). In response to SEB, we observed a stronger immunomodulant effect of cysteamine at 400 µM at both time points (**Supplementary Figure S2**, **Supplementary Table 1**). As IL-10 is crucial in the modulation of inflammation and acts as an anti-inflammatory factor (45, 46), we also evaluated the IL-10 production and found that, in response to PPD or SEB, IL-10 was significantly downmodulated by cysteamine in a dose-dependent manner after 6 h and 24 h, similarly to that observed for IL-2 (**Supplementary Figure S3**). This result indicates that cysteamine reduced both pro- and anti-inflammatory cytokines.

### Cysteamine reduces the percentage of lymphocytes in a dose- and time-dependent manner

**Figure 4:**
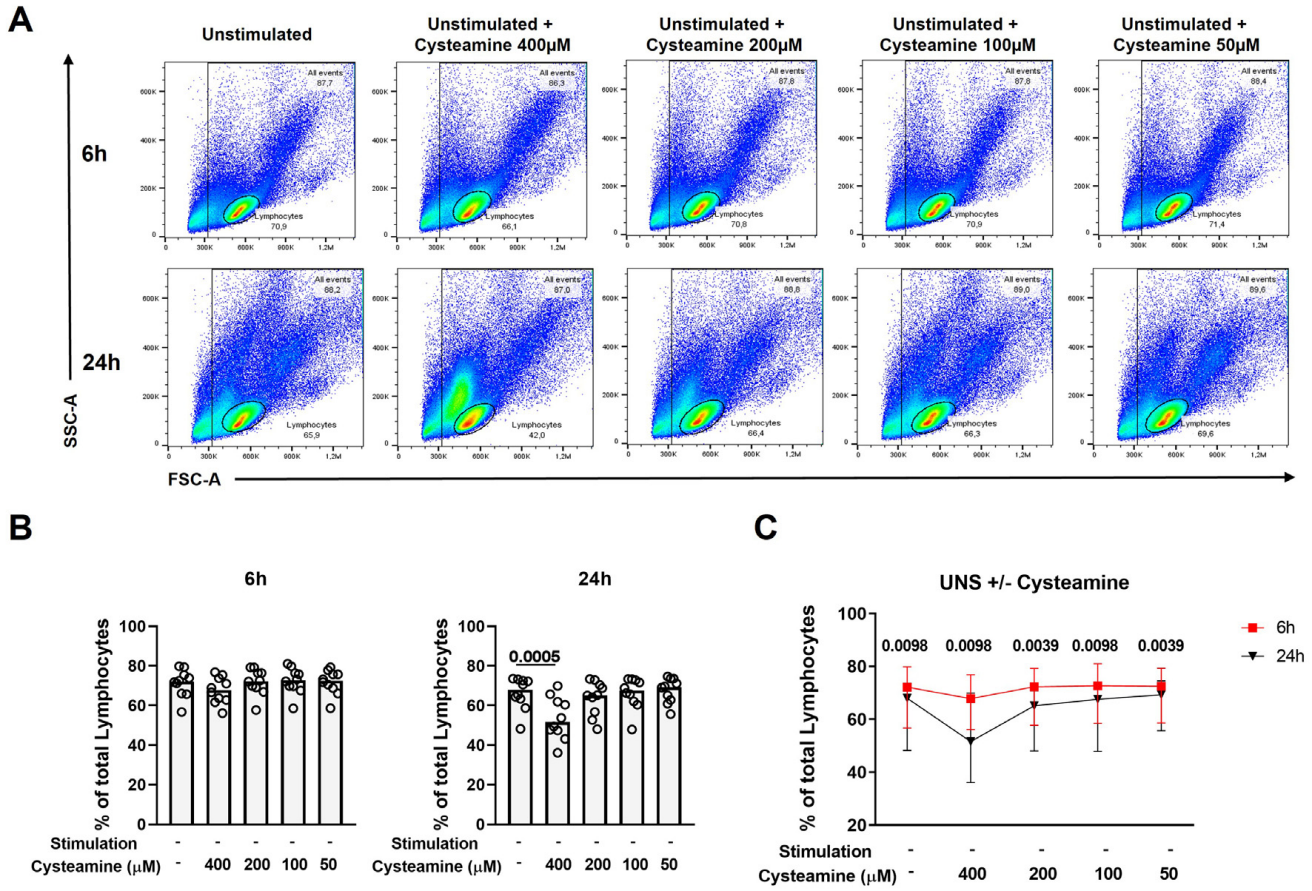
Cysteamine affects the percentage of lymphocytes in a dose- and time-dependent manner. Unstimulated PBMCs from 10 BCG-vaccinated HC were treated with cysteamine at different concentrations (50 µM–400 µM) for 6 hours (h) and 24 h. **(A)** The percentage of total lymphocytes was analyzed according to the morphological parameters (SSC-A and FSC-A) after 6 h (top panels) and 24 h (bottom panels). **(B)** Percentage of lymphocytes after cysteamine treatment for 6 h (left graph) or 24 h (right graph). **(C)** Comparison of the percentage of lymphocytes, 6 h red square and 24 h black triangle. Statistical analysis was performed using **(B)** Friedman test followed by Dunn’s multiple comparisons and **(C)** Wilcoxon matched-pair rank test. Data are expressed as median, and each dot represents a different subject.

While cysteamine at 400 µM significantly decreased the Th1 response, it concomitantly modified the lymphocyte cell morphology [evaluated as cellular complexity (SSC-A) and dimension (FSC-A) change], as highlighted by the flow cytometry analysis results (**Figure 4A**). Thus, we evaluated the potential *in vitro* cytotoxic effect of cysteamine using different concentrations (50 µM–400 µM) and different time points (6 h and 24 h) on the PBMCs of 10 BCG-vaccinated healthy controls. Treatment with cysteamine at 400 µM concentration for 24 h, but not for 6 h, significantly reduced the percentage of lymphocytes, gated as indicated in **Figure 4A** [68% (62.2%–72.7%) vs. 51.6% (46.3%–62.9%), p=0.0005; **Figure 4B** right panel]. Differently, cysteamine at lower concentrations did not significantly impact the percentage of the lymphocytes at any time point (**Figure 4B**). Comparing the two time points, a global significative reduction of the percentage of the lymphocytes was observed after 24 h of treatment; the utmost reduction occurred in cells treated with 400 µM of cysteamine (**Figure 4C**). Similar morphology results were observed in samples in which cysteamine was added to PPD or SEB stimuli (**Supplementary Figure S4**).

### Cysteamine induces cell death in a dose- and time-dependent manner

**Figure 5:**
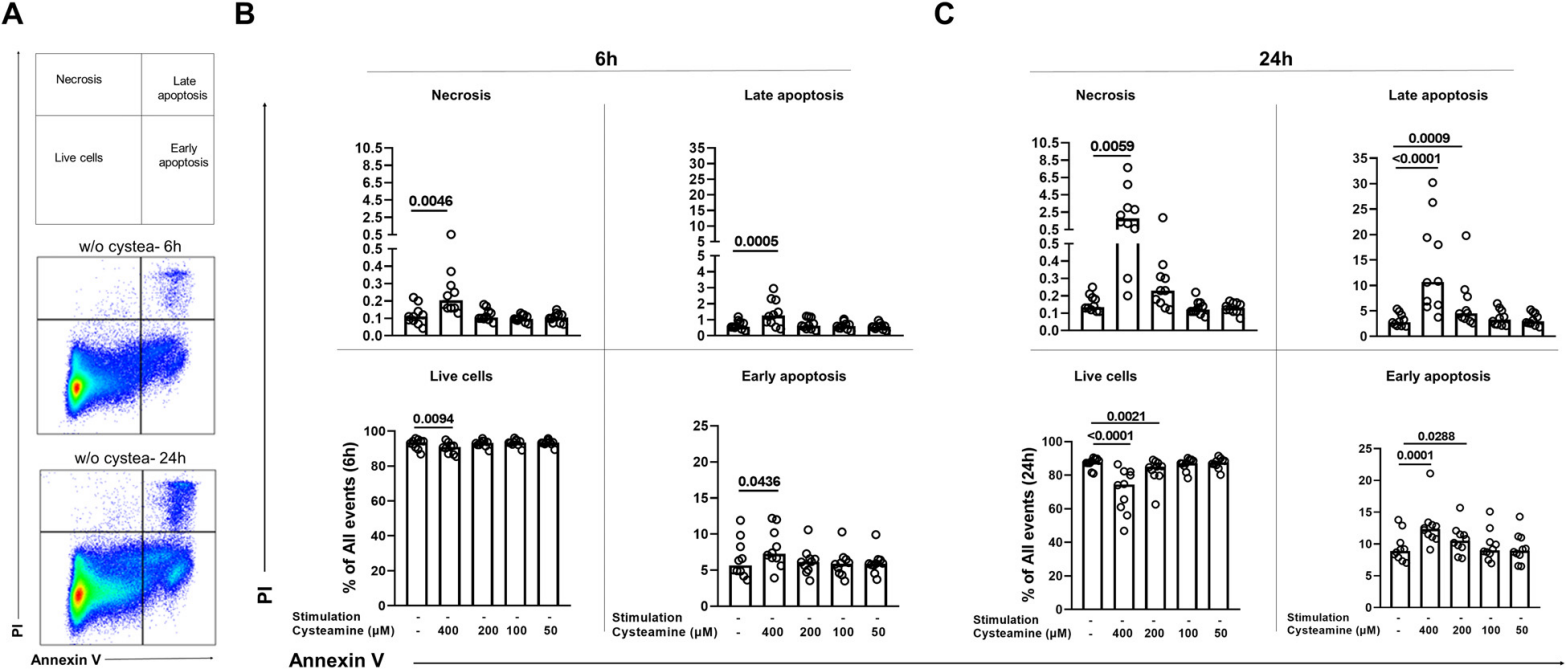
Analysis of apoptosis and necrosis in PBMCs treated with cysteamine at different concentrations and different time points by flow cytometry. Unstimulated PBMCs (BCG-vaccinated HC, n=10) were treated or not with cysteamine (50 µM–400 µM) for 6 h and 24 h. **(A)** All events acquired were gated, as schematically described at the top, after 6 h (middle) or 24 h (bottom) of incubation of unstimulated and untreated PBMCs as a representative gating strategy, based on the single or double positivity of Annexin V and/or PI. The apoptosis and necrosis were evaluated after **(B)** 6 h or **(C)** 24 h by flow cytometry. Statistical analysis was performed using Friedman test followed by Dunn’s multiple comparisons. Data are expressed as median, and each dot represents a single subject. PI, propidium iodide; w/o, without.

We then evaluated whether the morphological changes observed were dependent on apoptosis and/or necrosis induction. To this aim, we treated PBMCs from the 10 BCG-vaccinated healthy controls at different concentrations of cysteamine for either 6 h or 24 h and then stained them for Annexin V-FITC and Propidium Iodide-PE. Cells were gated as shown in **Figure 5A** (the complete gating strategy is shown **Supplementary Figure S5**). After 6 h of treatment, although statistically significant, cysteamine at 400 µM induced only less than 2% of early apoptosis when compared with the untreated sample (p=0.043), a <0.5% of increased in late apoptosis (p=0.0005), and a <0.1% of increased in necrosis (p=0.0046) (**Figure 5B**, **Supplementary Table 2**). Notably, after 24 h of cysteamine at 400 µM, PBMCs showed a significant increase in early apoptosis (p=0.0001), late apoptosis (p<0.0001), and necrosis (p=0.0059), indicating dose- and time-dependent effects. Moreover, at this time point, cysteamine at 200 µM significantly increased early (p=0.0288) and late (p=0.0009) apoptosis, although the differences in the percentage were very low (**Figure 5C**, **Supplementary Table 2**). We found similar results when cysteamine was added in the presence of either PPD or SEB regardless of the time of treatment (**Supplementary Figure S6**, **Supplementary Table 3**). Overall, these results indicate that cysteamine induced apoptosis and/or necrosis of PBMCs in a dose- and time-dependent manner.

## Discussion

In this study, we aimed to investigate whether cysteamine, in addition to its known antimicrobial effects against Mtb (25, 26), can influence the specific T-cell responses, as part of our exploration of host-directed therapies for infectious diseases. We show in human PBMCs that cysteamine decreases Th1 immune responses in PPD-specific or SEB-stimulated cells in a time- and dose-dependent manner, independently of endogenous IL-10. A decreased Tc1 response was also found after SEB stimulation. Finally, our data demonstrated that cysteamine at high doses induces late apoptosis and necrosis in a dose- and time-dependent manner. Altogether, these findings are important for both the scientific and clinical communities. For researchers, it is fundamental to understand the different effects of this drug on the immune system, particularly at concentrations commonly used for *in vitro* studies. For clinicians, it is critical to be aware of the immune modulatory effects of cysteamine and its potential use in HDT, especially in the context of ongoing and future clinical trials for infectious diseases such as TB and COVID-19 (26, 47).

Cysteamine may reduce cell inflammation through several mechanisms, for example inhibiting TG2. This may occur by generating a thiol-disulfide with a cysteine in the active site, thus preventing the enzyme’s transamidation functions or by acting as a TG2 substrate, thus resulting in a competitive inhibitor of the other amine substrate of this enzyme (48). TG2 is an enzyme known to be involved in the pathogenesis of several inflammatory conditions, such as allergic asthma (49), rheumatoid arthritis, fibrosis (50, 51), and infectious diseases including TB (25, 26). This is because TG2 activity is involved in regulating gene transcription by NF-кB activation either transamidating the NF-KB cellular inhibitor I-кB (52, 53) or by a transamidating-independent pathway (54). Therefore, the result of the present study, showing that cysteamine reduces Th1 and Tc1 responses mainly down-modulating IL-2, is likely due to NF-κB impairment functions (18). In pulmonary TB, the increased activity of TG2 in patients’ lungs may hinder macrophages from effectively killing Mtb (25). Interestingly, *in vitro* inhibition of TG2 by cysteamine treatment leads to antimycobacterial activity versus both Mtb and NTM in infected macrophages (55, 56). Altogether, this evidence supports that cysteamine may be an effective adjunct to antibiotic regimens for TB therapy, leading to a decreased bacterial load and inflammation due to its multiple capacities to inhibit TG2 and the NF-κB-mediated transcription of immune factors (25, 26, 57).

Despite its beneficial effects, cysteamine can be toxic at high concentrations, reducing cell proliferation and survival due to hydrogen peroxide (H_2_O_2_) production from its thiol group’s reaction with transition metals (18, 58) and the inhibition of glutathione peroxidase in several cell lines (18). In animal models (59–62), high doses of cysteamine induced apoptosis of duodenal epithelium dependent on cysteamine-mediated glutathione depletion, resulting in apoptosis-inducing factor (AIF) translocation (63). Here, we provide evidence that high doses of cysteamine impact also the viability of human PBMCs. The induction of apoptosis and necrosis was both dose- and time-dependent in unstimulated cells and even more pronounced in antigen-stimulated cells, suggesting a synergistic effect between antigenic stimulation and the effects of cysteamine at higher doses. Massive oxidative stress may have occurred in our experimental setting; however, we did not characterize the metabolic events, or the apoptotic signaling induced by cysteamine, as it was beyond the scope of this study. Moreover, it is reasonable to expect that these events may not be clinically relevant as the high cysteamine doses used in our study are far from the plasma concentrations of cysteamine reached during therapy in humans (64, 65). Indeed, the pharmacokinetics of cysteamine bitartrate under clinical treatment conditions was evaluated by administration of cysteamine bitartrate to cystinosis patients at their regular dose level in a single-dose, open-label, steady-state study and the mean ± SD plasma concentration for cysteamine was 36.3 ± 11.7 µM (range 16.9 µM–53.2 µM) (65). In the present study at this concentration, we did not find any significant apoptosis and or necrosis induced by cysteamine on PBMCs.

On the other hand, T-cell immune responses, and particularly Th1 cells, play a key role in TB as can control Mtb replication (4). Th1 cells are characterized by the production of pro-inflammatory cytokines including IL-2, IFN-γ, and TNF (66). TNF, a well-known proinflammatory cytokine produced mainly by monocytes, macrophages, and T cells, is involved in the inflammatory processes (67, 68) and, in TB, is crucial for the formation of a well-organized granuloma and host protection (69). However, its uncontrolled production can be harmful by activating a programmed necrosis (necroptosis) pathway and tissue damage (9, 68, 70, 71, 72). Similarly, while IFN-γ is an important mediator of a protective TB immune response, its excessive production can contribute to tissue damage. Therefore, it is important to find compounds that limit the harm related to an exaggerated immunity in several infectious diseases including TB and COVID-19. Indeed, we and others showed that cysteamine may down-modulate the hyper-inflammation caused by SARS-CoV-2 (13, 33, 34). Similarly, sustained inflammation may cause tissue damage in patients with TB resulting in permanent pulmonary disability. Thus, reducing excess inflammation by cysteamine may prevent lung damage and enhance the effectiveness of TB treatment. The clinical effects of cysteamine for TB treatment remain to be assessed by randomized, controlled, clinical trials.

The present study has limitations. While we showed that cysteamine has anti-inflammatory effects at low concentrations on PBMC from TB, TBI, and HC, we showed only in a group of healthy individuals that high doses of cysteamine may induce cell death. This is an interesting result, but it should be validated in TB patient cohorts in whom immune cells are already under an immune activation stress. Furthermore, a limited number of patients were included in this pilot study. Finally, the mechanisms leading to cellular damage were not studied in detail. Therefore, future studies, including a larger population of subjects, are needed to validate these results, and further characterize the cellular mechanisms involved in cysteamine-induced immunomodulation. Moreover, using various methods such as transcriptomics and multi-omics analysis or using appropriate animal models is required to reveal the exact mechanisms underlying these effects of cysteamine *in vivo*.

In conclusion, our results show that cysteamine reduces the Mtb-specific immune response whereas high doses induce late apoptosis and necrosis in a time- and dose-dependent manner. Overall, these findings could benefit the scientific community in designing experiments that assess the effects of cysteamine using *in vitro* studies. Additionally, these results highlight the potential of cysteamine as a valuable supplement to antimicrobial treatments, given its capacity to lower inflammation that may lead to tissue damage, commonly seen in TB and diseases like COVID-19.

## Data Availability

The raw data are available in our institutional repository (rawdata.inmi.it), subject to registration. The data can be found by selecting the article of interest from a list of articles ordered by year of publication. No charge for granting access to data is required. In the event of a malfunction of the application, the request can be sent directly by e-mail to the Library biblioteca@inmi.it.
