# Supplementary material for: Immunomodulatory effects of cysteamine and its potential use as a host-directed therapy for tuberculosis
Source: Front Immunol. 2024 Oct 28;15:1411827. doi: 10.3389/fimmu.2024.1411827 (PMC11550979; doi:10.3389/fimmu.2024.1411827)
Supplement: Supplementary file 1 [file DataSheet1.docx]

Supplementary Material

# Supplementary Tables

| **Supplementary Table 1: The effect of cysteamine on cytokines production evaluated by ELLA assay.** | | | | | | | | | | | | |
| --- | --- | --- | --- | --- | --- | --- | --- | --- | --- | --- | --- | --- |
|  |  | **PPD** | **PPD+ Cysteamine 400µM** | **p value^§^** | **PPD+**  **Cysteamine 200µM** | **p value^#^** | **SEB** | **SEB+**  **Cysteamine**  **400µM** | **p value^§^** | **SEB+ Cysteamine 200µM** | **p value^#^** |  |
| **6h** | IFN-γ | 26.3  (6.3-71.3) | 14.1  (3.8-29.3) | **0.0356** | 22.5  (5.0-66.4) | >0.9999 | 1153  (609.6-1938) | 869.3  (460.6-1895) | 0.2640 | 1168  (544.4-1555) | 0.1909 |  |
|  | TNF | 288.0  (197.7-443.1) | 346.7  (187.0-526.7) | >0.9999 | 299.0  (167.2-475.0) | >0.9999 | 1200  (869.7-1828) | 830.8  (575.2-1151) | **0.0028** | 1151  (757.2-1368) | 0.0648 |  |
|  | IL-2 | 25.0  (8.8-70.5) | 15.5  (4.4-27.3) | **0.0028** | 21.4  (4.0-59.1) | 0.0946 | 825.6  (495.2-951.5) | 484.4  (310.4-740.6) | **0.0075** | 666.1  (424.7-780.5) | 0.0946 |  |
| **24h** | IFN-γ | 140.7  (69-1273) | 197.1  (80.3-1318) | >0.9999 | 373.6  (92.1-1212) | >0.9999 | 10273  (9132-15062) | 7456  (4625-11431) | **0.0042** | 8054  (5847-14549) | 0.1014 |  |
|  | TNF | 274.9  (203.4-412.1) | 274.1  (136.3-306.4) | 0.0683 | 284.6  (164.8-348.8) | 0.7188 | 2565  (2138-3086) | 1910  (1649-2610) | **0.0115** | 2414  (1666-2915) | 0.4042 |  |
|  | IL-2 | 111.7  (41.2-317.7) | 41.6  (11.9-130) | **0.0001** | 75.3  (33.5-222.6) | **0.0292** | 2338  (2153-2956) | 1650  (1131-2097) | **0.0004** | 1930  (1539-2123) | **0.0451** |  |
| **Footnotes:** PPD: purified protein derivative of tuberculin; SEB: staphylococcal enterotoxin B; h: hours; IFN: interferon; TNF: tumor necrosis factor; IL: interleukin; §, # Friedman test with Dunn’s multiple correction between PPD (or SEB) vs PPD (or SEB) + Cysteamine 400µM and PPD (or SEB) vs PPD (or SEB) + Cysteamine 200µM, respectively. | | | | | | | | | | | | |

| **Supplementary Table 2: Cysteamine induced apoptosis and necrosis in time- and dose-dependent manner** | | | | | | |
| --- | --- | --- | --- | --- | --- | --- |
|  |  | **Unstimulated and untreated** | **Unstimulated + Cysteamine 400µM** | **p value^§^** | **Unstimulated + Cysteamine 200µM** | **p value^#^** |
| **6h** | % Live cells | 93.6  (90.4-94.6) | 90.9  (86.9-92.2) | **0.0094** | 93.2  (91.9-94.3) | 0.6292 |
|  | % Early apoptosis | 5.7  (4.7-8.6) | 7.2  (6.4-10.6) | **0.0436** | 6.2  (5.0-6.8) | >0.9999 |
|  | % Late apoptosis | 0.6  (0.5-0.9) | 1.3  (0.8-2.3) | **0.0005** | 0.6  (0.4-1.2) | 0.1909 |
|  | % Necrosis | 0.1  (0.08-0.15) | 0.2  (0.2-0.3) | **0.0046** | 0.1  (0.09-0.15) | >0.9999 |
| **24h** | % Live cells | 87.9  (85.9-89.9) | 74.4  (59.8-82.2) | **<0.0001** | 85.0  (79.9-87.6) | **0.0021** |
|  | % Early apoptosis | 8.9  (7.7-10.9) | 12.3  (11.0-13.1) | **0.0001** | 10.5  (9.1-11.6) | **0.0288** |
|  | % Late apoptosis | 2.8  (2.1-4.4) | 10.7  (6.1-21.1) | **<0.0001** | 4.5  (3.3-8.1) | **0.0009** |
|  | % Necrosis | 0.1  (0.1-0.2) | 1.8  (0.5-3.7) | **0.0059** | 0.2  (0.1-0.3) | 0.8124 |
| **Footnotes:** h: hours; §, # Friedman test with Dunn’s multiple correction between unstimulated and untreated vs unstimulated + Cysteamine 400µM and unstimulated and untreated vs unstimulated + Cysteamine 200µM, respectively | | | | | | |

| **Supplementary Table 3: Cysteamine induced apoptosis and/or necrosis in PPD- and SEB- stimulated PBMCs.** | | | | | | | | | | | | |
| --- | --- | --- | --- | --- | --- | --- | --- | --- | --- | --- | --- | --- |
|  |  | **PPD** | **PPD+ Cysteamine 400µM** | **p value^§^** | **PPD+**  **Cysteamine 200µM** | **p value^#^** | **SEB** | **SEB+**  **Cysteamine**  **400µM** | **p value^§^** | **SEB+ Cysteamine 200µM** | **p value^#^** |  |
| **6h** | % Live cells | 95.5  (94.0-96.4) | 92.9  (87.5-94.4) | **0.0036** | 95.2  (91.8-95.9) | 0.2250 | 91.6  (90.1-92.1) | 87.2  (83.6-91.1) | **0.0003** | 89.7  (87.8-91.5) | **0.0436** |  |
|  | % Early apoptosis | 3.8  (3.1-5.2) | 5.9  (4.3-10.8) | **0.0094** | 4.0  (3.3-6.7) | 0.4792 | 7.4  (6.5-8.2) | 10.0  (7.5-13.5) | **0.0028** | 8.7  (6.9-9.6) | 0.1356 |  |
|  | % Late apoptosis | 0.5  (0.3-0.6) | 0.9  (0.6-2.3) | **<0.0001** | 0.6  (0.4-1.2) | 0.0785 | 1.2  (0.8-1.6) | 2.2  (1.6-3.0) | **<0.0001** | 1.7  (1.0-1.9) | 0.0005 |  |
|  | % Necrosis | 0.1  (0.09-0.1) | 0.3  (0.2-0.4) | **0.0036** | 0.1  (0.1-0.2) | 0.2640 | 0.2  (0.1-0.2) | 0.4  (0.2-0.5) | **0.0007** | 0.2  (0.2-0.3) | **0.0233** |  |
| **24h** | % Live cells | 90.2  (89.2-93.9) | 77.2  (63.0-82.8) | **<0.0001** | 88.2  (83.5-90.9) | **0.0021** | 84.6  (82.9-86.0) | 71.0  (52.7-75.7) | **0.0003** | 81.6  (78.8-83.7) | 0.0648 |  |
|  | % Early apoptosis | 7.3  (4.9-9.3) | 8.5  (6.4-11.5) | **0.0003** | 8.4  (5.7-10.3) | 0.1356 | 9.4  (7.3-10.2) | 10.9  (8.8-11.9) | 0.0533 | 9.6  (6.5-10.6) | 0.7164 |  |
|  | % Late apoptosis | 1.2  (1.1-1.8) | 10.2  (5.0-22.9) | **<0.0001** | 1.9  (1.7-9.3) | **0.0046** | 6.1  (4.5-7.1) | 15.7  (10.7-28.3) | **<0.0001** | 8.7  (7.2-10.7) | **0.0009** |  |
|  | % Necrosis | 0.1  (0.1-0.2) | 3.8  (0.8-8.3) | **<0.0001** | 0.3  (0.2-1.2) | **0.1135** | 0.3  (0.2-0.6) | 3.4  (1.5-6.6) | **<0.0001** | 0.4  (0.4-1.1) | 0.1356 |  |
| **Footnotes:** PPD: purified protein derivative of tuberculin; SEB: staphylococcal enterotoxin B; h: hours; §, # Friedman test with Dunn’s multiple correction between PPD (or SEB) vs PPD (or SEB) + Cysteamine 400µM and PPD (or SEB) vs PPD (or SEB) + Cysteamine 200µM, respectively. | | | | | | | | | | | | |

# Supplementary Figures

**Supplementary Figure S1**


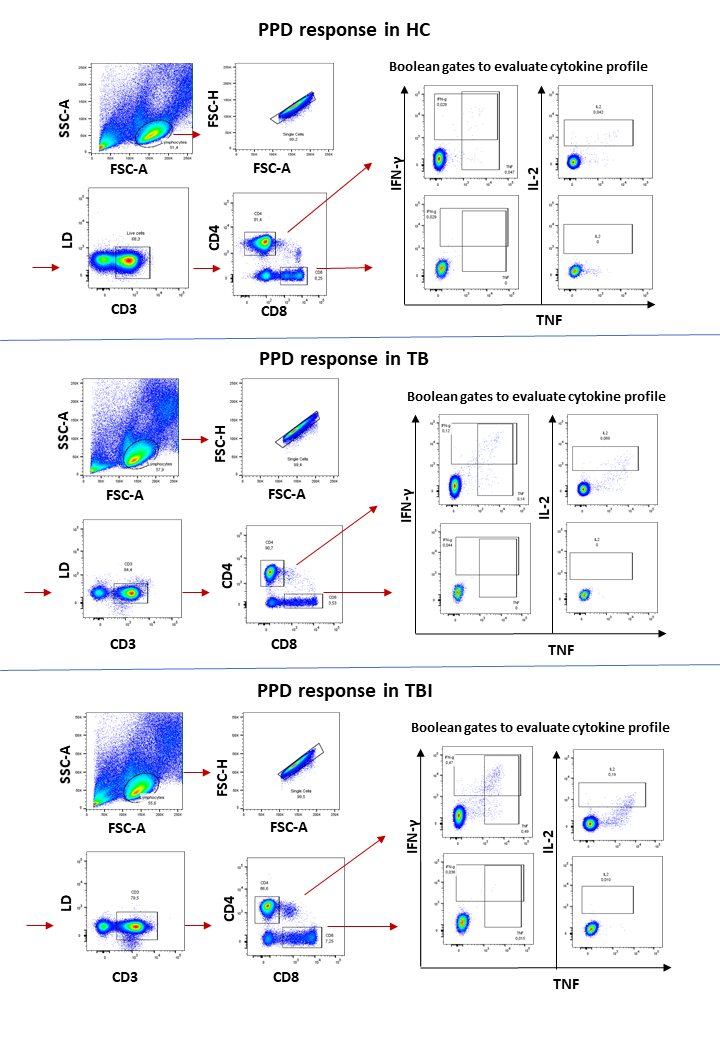


**Supplementary Figure S1**. Flow cytometry gating strategy for Th1 and Tc1 cytokine production. PBMCs were stained with surface markers and then intracellular cytokine staining was performed as described in the methods section in HC, TB, and TBI individuals, unstimulated (UNS) or stimulated with PPD or SEB and treated or not with cysteamine. The gating strategy shown is representative of a subject of each group stimulated with PPD.

**Supplementary Figure S2**

**
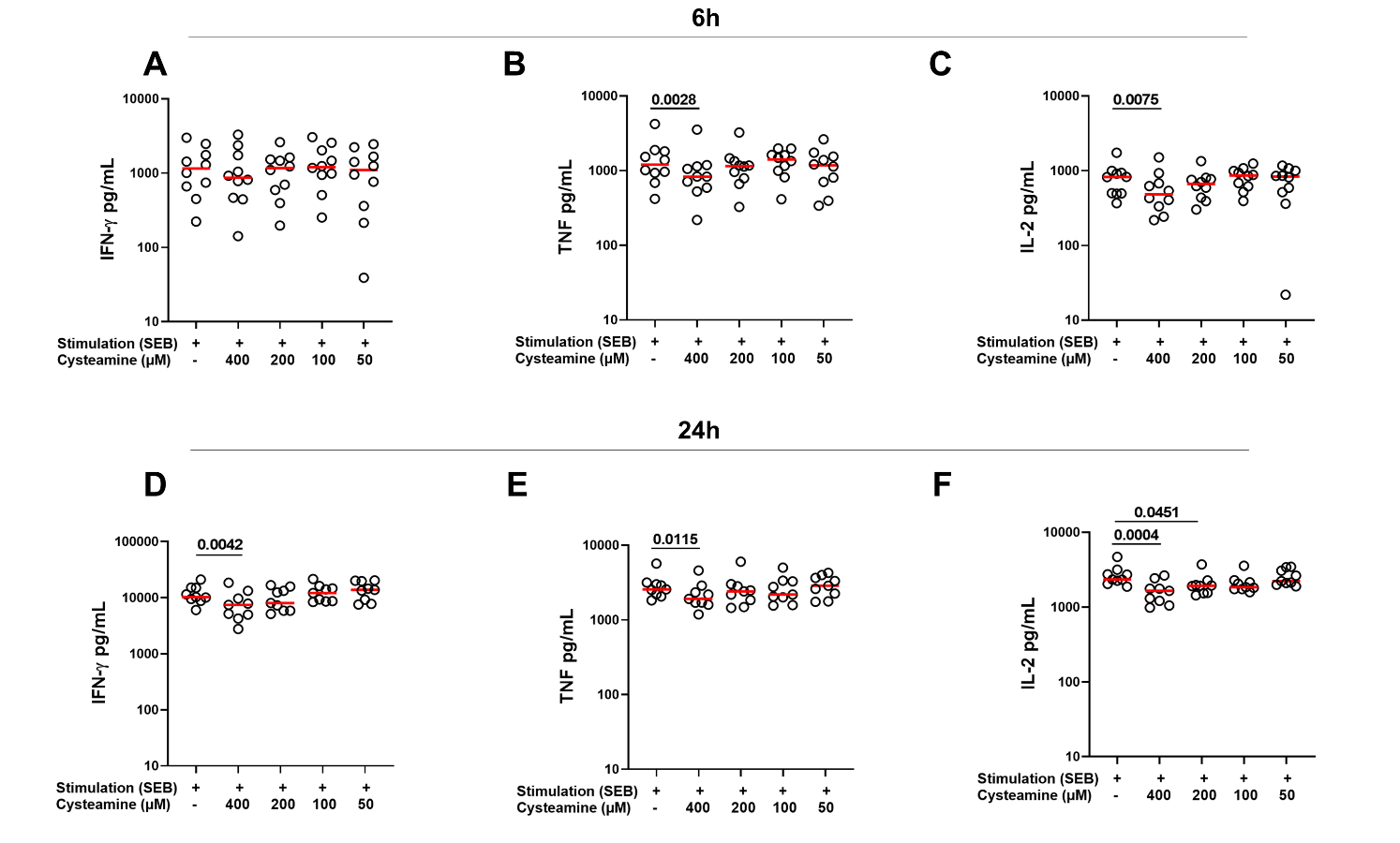
**

**Supplementary Figure S2. Cytokines production in response to SEB.** Levels of cytokines were evaluated in supernatants of PBMCs (BCG-vaccinated HC n=10) stimulated with SEB for 6h (**A-C**) and 24h (**D-F**) and treated or not with cysteamine (50µM-400µM). (**A, D**) IFN-γ, (**B, E**) TNF and (**C, F**) IL-2 were measured using an automated ELISA assay (ELLA). Values from stimulated samples were subtracted from the respective unstimulated control. Statistical analysis was performed using Friedman test followed by Dunn’s multiple comparisons. Red lines indicated the median and each dot represents a different individual.

**Supplementary Figure S3**

**
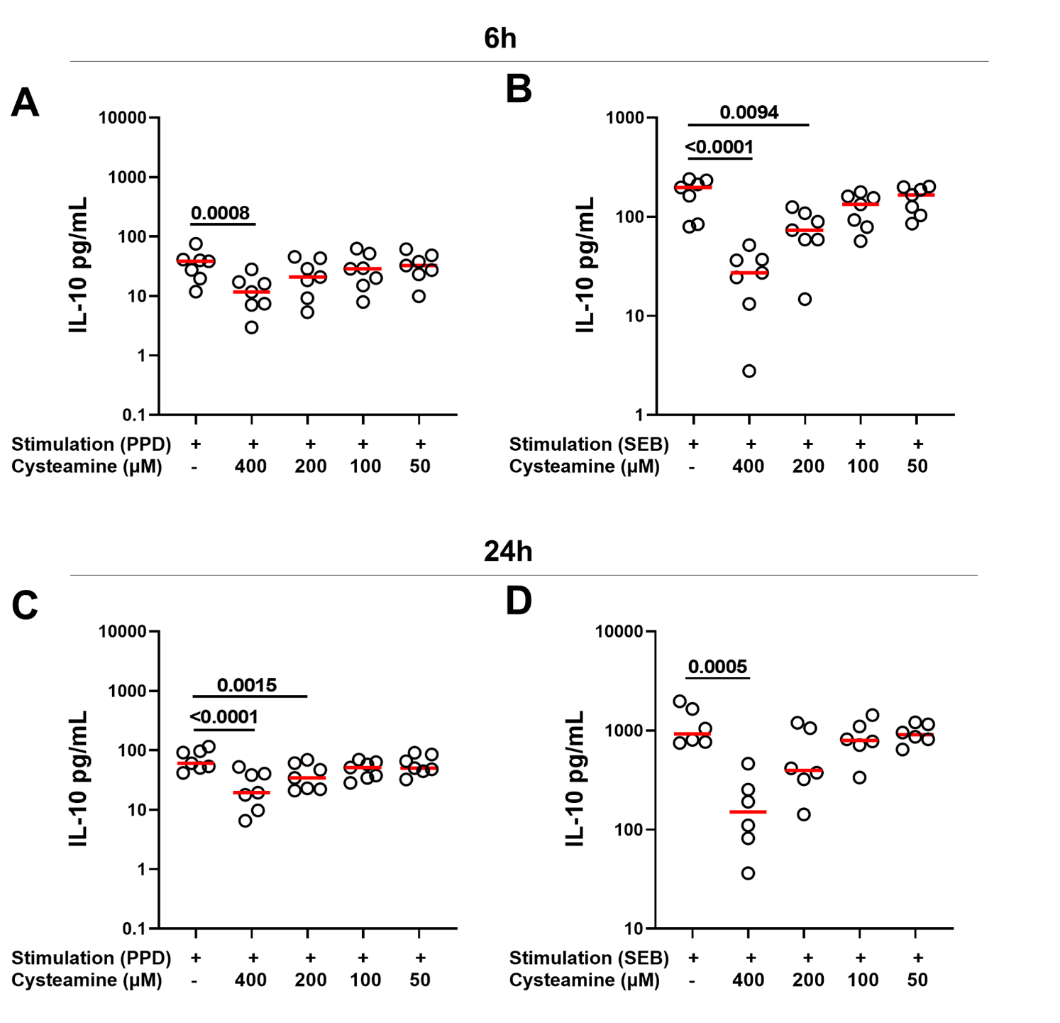
**

**Supplementary Figure S3. IL-10 production in response to PPD or SEB.** Level of IL-10 was evaluated in supernatants of PBMCs (BCG-vaccinated HC n=7) stimulated with PPD or SEB for 6h (**A-B**) and 24h (**C-D**) and treated or not with cysteamine (50µM-400µM) using ELLA, an automated ELISA assay. Values from stimulated samples were subtracted from the respective unstimulated control. Red lines indicated the median and each dot represents a different individual. Statistical analysis was performed using Friedman test followed by Dunn’s multiple comparisons.

**Supplementary Figure S4**

**
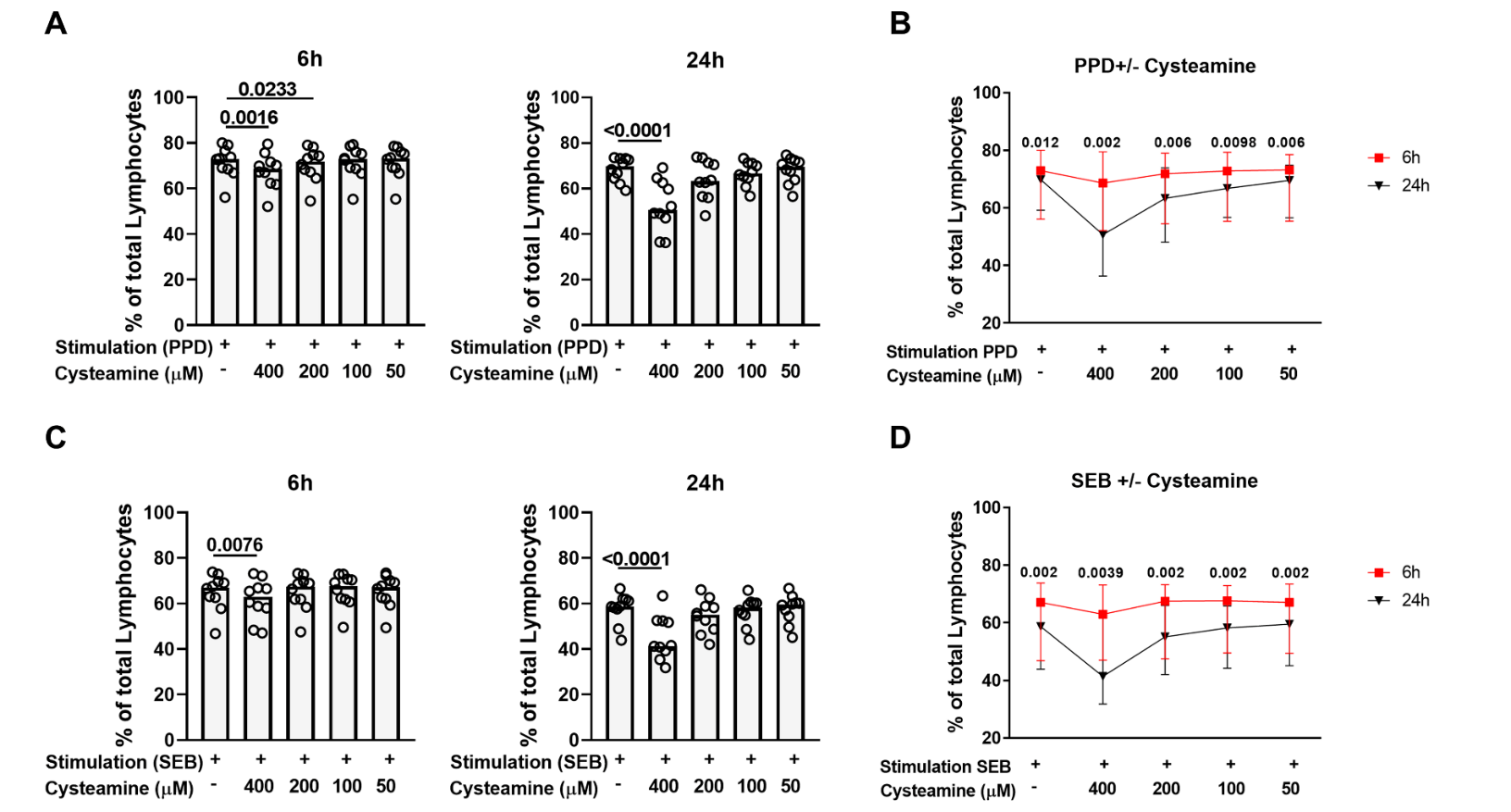
**

**Supplementary Figure S4. Cysteamine affects the percentage of lymphocytes in a dose- and time-dependent manner, independent of stimuli.** PBMCs from 10 BCG-vaccinated HC were stimulated *in vitro* with PPD (**A, B**) or SEB (**C, D**) and treated with cysteamine (400µM, 200µM, 100µM and 50µM) for 6 hours (h) and 24 h. Samples were acquired after 6h (A) and (C) 24h and lymphocytes were gated. (**B, D)** Graph reported the percentage of lymphocytes at both time points (6h, red square; 24h, black triangle). Statistical analysis was performed using (B) Friedman test followed by Dunn’s multiple comparisons and (C) Wilcoxon matched-pairs rank test. Data are expressed as median and each dot represents a different individual.

**Supplementary Figure S5**
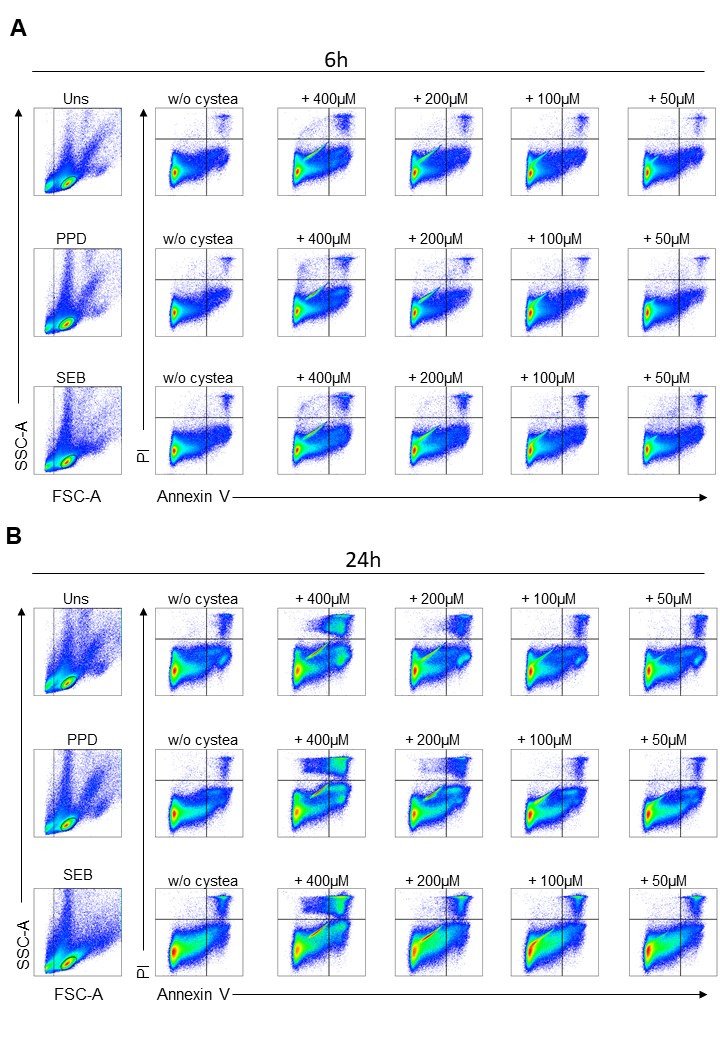


**Supplementary Figure S5. Flow cytometry gating strategy for AnnexinV/PI.** PBMCs from 10 BCG-vaccinated HC were stained with Annexin V and PI according to manufacturer’s instruction and total cells were gated as described in (A) after 6h and (B) after 24 h. Samples were left unstimulated (UNS) or stimulated with PPD or SEB and treated or not with cysteamine at different concentrations (+ 400µM, + 200µM, + 100µM, + 50µM). The gating strategy shown is representative of a healthy control (HC).

**Supplementary Figure S6**

**
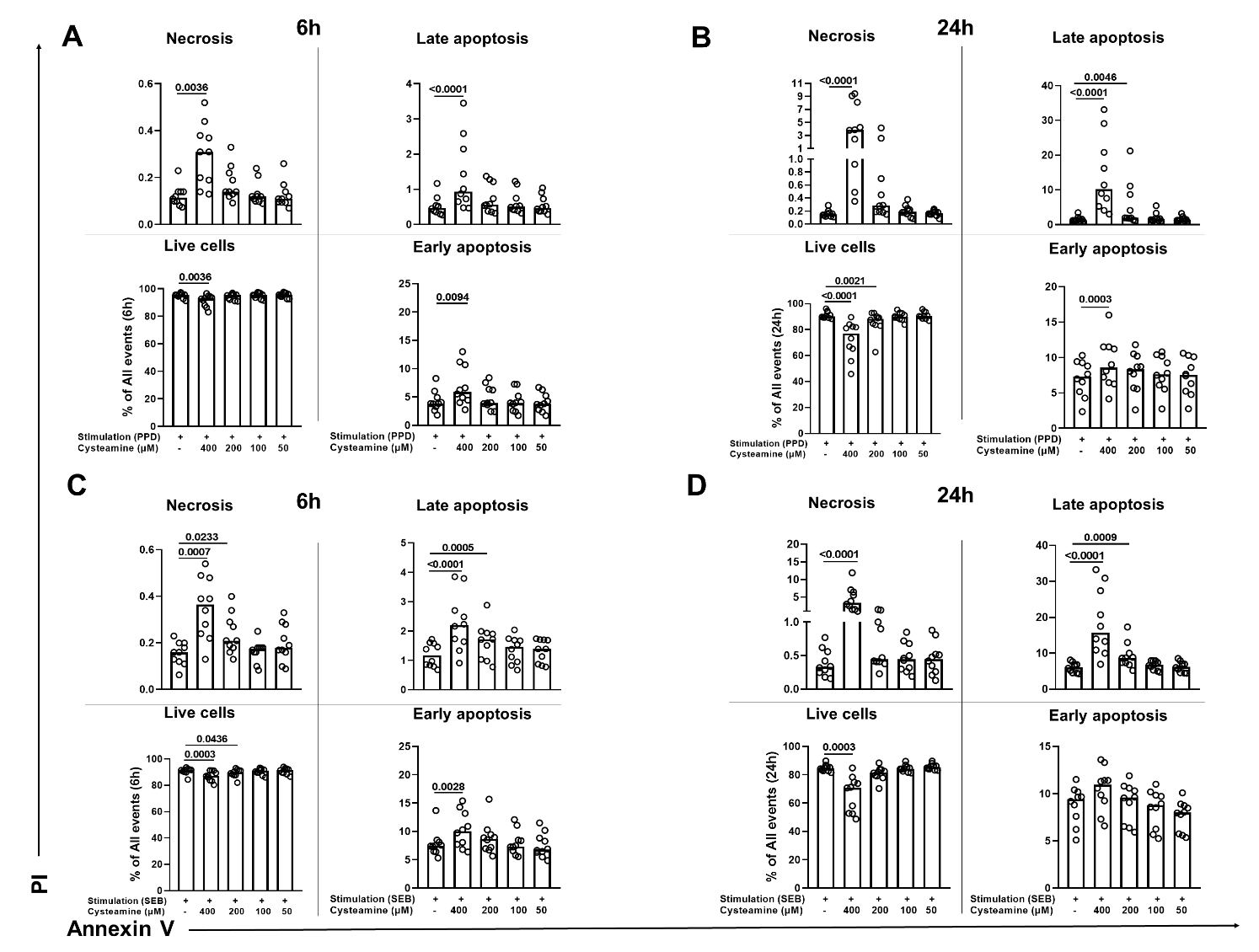
**

**Supplementary Figure S6. Analysis of apoptosis and necrosis in stimulated PBMCs treated or not with cysteamine at different concentrations and different time points by flow cytometry.** PBMCs from 10 BCG-vaccinated HC were treated or left untreated with cysteamine at different concentrations and stimulated for 6h (**A, C**) and 24h (**B, D**) with PPD (**A, B**) or SEB (**C, D**). The all events acquired were gated as described in figure 2A and the percentage of cells that were in apoptosis and necrosis were evaluated by flow cytometry. Statistical analysis was performed using Friedman test followed by Dunn’s multiple comparisons. Data are expressed as median and each dot represents a different subject. PI, Propidium iodide.
